# Wet Bulb Globe Temperature: Indicating Extreme Heat Risk on a Global Grid

**DOI:** 10.1029/2022GH000701

**Published:** 2023-02-20

**Authors:** Chloe Brimicombe, Chun Hay Brian Lo, Florian Pappenberger, Claudia Di Napoli, Pedro Maciel, Tiago Quintino, Rosalind Cornforth, Hannah L. Cloke

**Affiliations:** ^1^ Department of Geography and Environmental Science University of Reading Reading UK; ^2^ European Centre for Medium‐Range Weather Forecasts (ECMWF) Reading UK; ^3^ Walker Institute University of Reading Reading UK; ^4^ Department of Meteorology University of Reading Reading UK; ^5^ School of Agriculture, Policy and Development University of Reading Reading UK; ^6^ Department of Earth Sciences Uppsala University Uppsala Sweden; ^7^ Centre of Natural Hazards and Disaster Science CNDS Uppsala Sweden

**Keywords:** heatwaves, heat stress, WBGT, mean radiant temperature, climate change and health, numerical weather prediction

## Abstract

The Wet Bulb Globe Temperature (WBGT) is an international standard heat index used by the health, industrial, sports, and climate sectors to assess thermal comfort during heat extremes. Observations of its components, the globe and the wet bulb temperature (WBT), are however sparse. Therefore WBGT is difficult to derive, making it common to rely on approximations, such as the ones developed by Liljegren et al. (2008, https://doi.org/10.1080/15459620802310770, ${WBGT}_{Liljegren}$) and by the American College of Sports Medicine (${WBGT}_{ACSM87}$). In this study, a global data set is created by implementing an updated WBGT method using ECMWF ERA5 gridded meteorological variables and is evaluated against existing WBGT methods. The new method, ${WBGT}_{Brimicombe}$, uses globe temperature calculated using mean radiant temperature and is found to be accurate in comparison to ${WBGT}_{Liljegren}$ across three heatwave case studies. In addition, it is found that ${WBGT}_{ACSM87}$ is not an adequate approximation of WBGT. Our new method is a candidate for a global forecasting early warning system.

## Key Points

- We create an accurate method for calculating Wet Bulb Globe Temperature (WBGT) using Mean Radiant Temperature termed WBGT_Brimicombe_
- It is found that WBGT_amsc87_ also known as WBGTsimple is not an accurate approximation of WBGT
- WBGT_Brimicombe_ can assist with robust heat stress standards across sectors including in public and occupational health

## 1 Introduction

The Wet Bulb Globe Temperature (WBGT) is an International Standards Organisation (ISO) approved metric of heat stress in humans (Int Org Standard, 2017). Heat stress is caused by the build‐up of body heat either as a result of exertion and/or exposure to the external environment (air temperature humidity, solar radiation, wind speed etc.) (D’Ambrosio Alfano et al., 2014; Ioannou et al., 2022; Jacklitsch et al., 2016; McGregor & Vanos, 2018; Parsons, 2006). WBGT was originally developed in the 1950s as part of a campaign to lower the risk of heat disorders during the training of US Army and Marine troops (Minard, 1961).

The WBGT has many applications and is used widely in many research areas such as the occupational and public health sectors. In addition, it is used in the sports and exercise field, industrial hygiene and in climate change research and is one of the most popular heat stress indices (Heo et al., 2019; Kjellstrom et al., 2009; Lemke & Kjellstrom, 2012; Lucas et al., 2014; Racinais et al., 2015).

The WBGT (°C) is defined by three environmental variables via the following equation (Minard, 1961):

(1) $$WBGT={0.7T}_{w}+0.2T_{g}+0.1T_{a}$$

where $T_{a}$ is 2 m air temperature (i.e., dry bulb temperature, in °C), $T_{g}$ is globe thermometer temperature (°C), and $T_{w}$ is natural wet bulb thermometer temperature (°C).

Whereas 2 m air temperature is easily measurable, observations of globe thermometer and wet bulb thermometer temperatures are often sparse (Budd, 2008; D’Ambrosio Alfano et al., 2014). Consequently, it has been historically challenging to calculate WBGT from Equation 1 and it is instead common to rely on approximations. These include the approximation from the American College of Sports Medicine (termed ${WBGT}_{ACSM87}$), which is a linear model of the WBGT (American college of sports medicine, 1987), and the approximation by Liljegren and colleagues (termed ${WBGT}_{Liljegren}$), which is a more complex approximation based on the fundamentals of heat transfer (Liljegren et al., 2008).

In this study, we compare a new approach to approximate WBGT (termed ${WBGT}_{Brimicombe}$) with ${WBGT}_{ACSM87}$ and ${WBGT}_{Liljegren}$. Our approach is novel in calculating WBGT from gridded data using the variable of mean radiant temperature and is designed for operational forecasting systems. Comparisons are performed globally by using the ERA5 hourly global gridded reanalysis from the European Centre for Medium‐Range Weather Forecasts (ECMWF), Observation data from the World Radiation Monitoring Center–Baseline Surface Radiation Network (Driemel et al., 2018) and are here discussed within the context of three heatwave case studies (India and Pakistan in July 2003, the Western Sahel in March 2013 and Australia in December 2019).

## 2 Method

### 2.1 Brimicombe WBGT Approximation (WBGT_Brimicombe_)

This new approach to approximate WBGT has been developed for numerical weather prediction post‐processing as it takes an optimized approach to the calculation of WBGT by removing the need for iterative loops. We calculate globe temperature using an adapted version of the original Bedford and Warner equation, making use of mean radiant temperature, a measurement of incidence of radiation on a body which is appropriate for indoor or outdoor use depending on given inputs (Bedford & Warner, 1934; De Dear, 1987; Guo et al., 2018; Thorsson et al., 2007; Vanos et al., 2021).

Here Equation 2 is used to solve for globe temperature as the subject because the ERA5 reanalysis data contains the variables of 2 m air temperature (*T*_a_), 10‐m wind speed (*v*_a_), and mean radiant temperature (*T*_MRT_). All temperatures are in Kelvin; 10‐m wind speed was found to be within ±1°C of an approximated 2 m wind speed which used the method found in Spangler et al. (2022) and therefore is used (not shown). The code to compute this is available as part of thermofeel: https://doi.org/10.21957/mp6v-fd16 (Brimicombe et al., 2021; Brimicombe, Di Napole et al., 2022)

(2) $$T_{MRT}=\sqrt[{4}]{{T_{g}}^{4}+\frac{h_{cg}}{\varepsilon \times D^{0.4}}\times \left(T_{g}-T_{a}\right)}$$

In Equation 2, $h_{cg}$ is the mean convection coefficient and is calculated using Equation 3. This is an additional correction from the original method and reduces the impact weighting of high wind speeds on the outputted globe temperature (De Dear, 1987; Guo et al., 2018).

(3) $$h_{cg}=1.1\times 10^{8}\times {v_{a}}^{0.6}$$

To calculate the wet bulb temperature (WBT), a theoretical method by Stull (2011) is used and is shown in Equation 4, where *T*_a_ is 2 m air temperature in °C and RH is relative humidity in percent. This method is valid between −20°C and 50°C and between 5% and 99% humidity, which are the ranges the method is optimized for and with which it has been used in previous studies (Freychet et al., 2020; Heo et al., 2019; Raymond et al., 2017). In addition, this method provided a test case, an expected value for a given set of inputs, which allowed validation of the calculated value (Stull, 2011).

(4) $$\begin{array}{ccc} T_{w} & = & T_{a}\;\tan^{-1}{(0.151977\left(RH+8.313659\right)}^{1/2})\\ & & +\tan^{-1}\left(T_{a}+RH\right)\\ & & -\tan^{-1}\left(RH-1.676331\right)+{0.00391838\left(RH\right)}^{3/2}\\ & & \times \tan^{-1}\left(0.023101\times RH\right)-4.686035 \end{array}$$

Once calculated, the globe temperature and WBT along with 2 m air temperature are used in Equation 1 to provide the ${WBGT}_{Brimicombe}$.approximation.

### 2.2 Liljegren WBGT Approximation (WBGT_Liljegren_)

WBGT_Liljegren_ can be considered a existing “gold standard” benchmark WBGT value as it is widely considered the most accurate WBGT approximation available (Kjellstrom et al., 2009; Kong & Huber, 2021; Liljegren et al., 2008). To obtain WBGT_Liljegren_, WBT is calculated as per Equation 5 and globe temperature is calculated as per Equation 6 which are then used in Equation 1. Specifically, WBT is calculated as

(5) $$T_{w}=T_{a}-Nu\times Sh\times {\left(\frac{\mathrm{Pr}}{Sc}\right)}^{a}\left(\frac{e_{w}-e_{a}}{P-e_{w}}\right)+\frac{∆F_{net}}{A_{h}}$$

where Nu is the Nusselt number, Sh is the Sherwood Number, Pr is the Prandtl number, and Sc is the Schmidt number and $\left(\frac{e_{w}-e_{a}}{P-e_{w}}\right)$ is the change in saturation water vapor transfer between the hygrometer wick and its surroundings. $\frac{∆F_{net}}{A_{h}}$ is the net radiative heat flux divided by the convective heat transfer coefficient. Full details can be seen in (Liljegren et al., 2008). Globe temperature is calculated as

(6) $$T_{g}^{4}=\frac{1}{2}\left(1+\varepsilon_{a}\right)T_{a}^{4}-\frac{h}{\varepsilon_{g}\sigma }\left(T_{g}-T_{a}\right)+\frac{ssrd}{2\varepsilon_{g}\sigma }\left(1-\alpha_{g}\right)\left[1+\left(\frac{1}{2\;\cos\;\theta }\right)dsrp+\alpha_{sfc}\right]$$

where the $\frac{h}{\varepsilon_{g}\sigma }\left(T_{g}-T_{a}\right)\;and\;\frac{S}{2\varepsilon_{g}\sigma }\left(1-\alpha_{g}\right)$ terms denote the energy gain from diffuse downwards and direct downward solar radiation respectively. dsrp is the projected area and $\alpha_{sfc}$ is the reflected solar radiation and ssrd is downward solar radiation. Full details can be seen in Liljegren et al. (2008) . Here data for WBGT_Liljegren_ is provided by Kong and Huber (2021), instead of calculation using the HEAT‐SHIELD methodology, because the method presented by Kong and Huber appears to be more robust and closer to the original methodology (Casanueva, 2017).

### 2.3 American College of Sports Medicine WBGT Approximation (WBGT_ACSM87_)

The ${WBGT}_{ACSM87}$ (American college of sports medicine, 1987) was also calculated (Equation 7) as it continues to appear widely in literature, despite it being known to have large bias (Chen et al., 2019; Grundstein & Cooper, 2018; Kong & Huber, 2021). ${WBGT}_{ACSM87}$ is calculated from 2 m air temperature and saturation water vapor pressure (e) as:

(7) $${WBGT}_{ACSM87}=0.567\times T_{a}+0.393\times e+3.94$$

### 2.4 Methodological Difference Between WBGT_Brimicombe_ and WBGT_Liljegren_

Several methodological differences between our new WBGT approximation and the existing “gold standard” WBGT approximation are present (Brimicombe, Di Napoli et al., 2022; Liljegren et al., 2008).

One key variable that is necessary in the calculation of *T*_g_ (both in Equations 2 and 6) is the cosine of the solar zenith angle. In previous studies it is found that radiation in the Liljegren *T*_g_ methodology has inaccuracies at sunrise and sunset due to the method used to calculate this variable (Kong & Huber, 2022; Lemke & Kjellstrom, 2012). These inaccuracies are known to become greater in a numerical weather prediction service time step (a period of several hours) (Brimicombe, Quintino, et al., 2022; Hogan & Hirahara, 2016). For the Brimicombe *T*_g_ methodology this does not occur because a specially designed cosine of the solar zenith angle is implemented (Brimicombe, Di Napoli et al., 2022; Brimicombe, Quintino, et al., 2022).

Another difference is in the number of radiation input variables used to calculate *T*_g_ in the Liljegren methodology. In this only 2 radiation components are used (Liljegren et al., 2008) in comparison to the 5 that calculate *T*_MRT_ (please refer to: Di Napoli et al., 2020) which goes on to calculate the Brimicombe *T*_g_. Equation 2, which expresses mean radiant temperature *T*_MRT_ as a function of *T*_g_ and *T*_a_, is comparable to the heat balance expressed in Bedford and Warner (1934), therefore relating mean radiant temperature to the temperature of surrounding surfaces. Similarly, Equation 2 can also be rearranged in order of *T*_g_^4^, where many comparable terms to Equation 6 are identifiable.

In the Liljegren *T*_w_ methodology a psychrometric WBT is calculated using fundamentals of mass transfer. In addition a key input of saturation water vapor pressure is calculated differently over ice and water (and the land surface) as in Hardy (1998). In comparison, in the Brimicombe *T*_w_ methodology an empirical theoretical WBT is calculated (Stull, 2011). As previously mentioned, this computationally removes the need for iterative loops, which are onerous to run for a gridded data set. In addition, the saturation water vapor pressure method is only for over water (and the land surface) in contrast to being over either water or ice, given that WBGT is a human heat stress index. How these methodological differences introduce errors will be explored within this study.

### 2.5 WBGT Approximation Comparisons

To compare the WBGT approximations, this study uses variables available as part of the ERA5 and ERA5‐HEAT gridded reanalysis data sets produced by ECMWF on a 0.25° × 0.25° grid at an hourly time step (Di Napoli et al., 2021; Hersbach et al., 2020). ERA5 was chosen for this study as a state‐of‐the‐art gridded reanalysis data set; it is an ideal data set to test out a new gridded based methodology and has the added benefit of outputting the mean radiant temperature variable (the incidence of radiation on the body). ERA5 and ERA5‐HEAT variables of 2 m air temperature, 2 m dew point temperature, 10 m wind speed and mean radiant temperature are used in the relevant equations to calculate ${WBGT}_{Brimicombe}$ and ${WBGT}_{ACSM87}$. ${WBGT}_{Liljegren}$ is also calculated using ERA5 reanalysis data. There are known limitations of ERA5; these include inaccuracies at higher elevations (Brunamonti et al., 2019; Senyunzi et al., 2020).

The approximations are calculated and compared for three past heatwaves on dates where heat stress is known to have occurred. One affected India and Pakistan in July 2003, another the Western Sahel in March 2013 and another Australia in December 2019 (CRED, 2020). In addition, this study also considers the full global gridded data sets of WBGT values including those below a heat stress threshold.

The gridded ERA5 reanalysis output for ${WBGT}_{Brimicombe}$ is compared to the observed ${WBGT}_{Brimicombe}$ calculated using data from the Tateno TAT (36.1°N, 140°E) and Nya Långenäs NYA (78.9°N, 11°E) stations from the World Radiation Monitoring Center–Baseline Surface Radiation Network (WRMC–BSRN, Driemel et al., 2018) for Daily Maximum WBGT for July 2003 and March 2013. In addition, for March 2013 the Stull WBT method is compared to the Davies‐Jones method (available at: https://github.com/smartlixx/WetBulb/blob/master/WetBulb.py) (Davies‐Jones, 2008; Stull, 2011). We are constrained by the available observation data.

The comparison between the three approximations uses the observed WBGT thresholds set out by the ISO (Jacklitsch et al., 2016; Table 1). According to ISO, which considers heat stress by reference to recommended lifting and hard labor workloads, 33°C is known as a critical health threshold for WBGT (Heo et al., 2019).

**Table 1:** Heat Stress Thresholds for Wet Bulb Globe Temperature and Recommended Labor Legal Definitions of Workloads for an Average Period of Work

WBGT (°C)	Recommended maximum workload	Approximated work/rest cycles (minutes)	Category
>33	Resting	Rest	5
30–33	Light	15/45	4
28–30	Moderate	30/30	3
25–28	Heavy	30/15	2
23–25	Very heavy	45/15	1
<23	No recommendations	No recommendations	0

*Note*. Adapted from Jacklitsch et al. (2016).

${WBGT}_{ACSM87}$ and ${WBGT}_{Brimicombe}$ are compared against the existing gold standard approximation of ${WBGT}_{Liljegren}$ in two ways. The first is through evaluation of the spatial anomaly in WBGT values. ${WBGT}_{Liljegren}$ is subtracted from the corresponding ${WBGT}_{ACSM87}$ or ${WBGT}_{Brimicombe}$ values. Second, is a correlation between ${WBGT}_{Liljegren}$ and the other WBGT approximations is assessed together with the mean absolute error (MAE). In addition the sensitivity of the outputted ${WBGT}_{Brimicombe}$ and ${WBGT}_{Liljegren}$ approximations to key input variables is assessed.

## 3 Results

### 3.1 Gridded Outputs of WBGT for the Three Heatwaves

**Figure 1:**
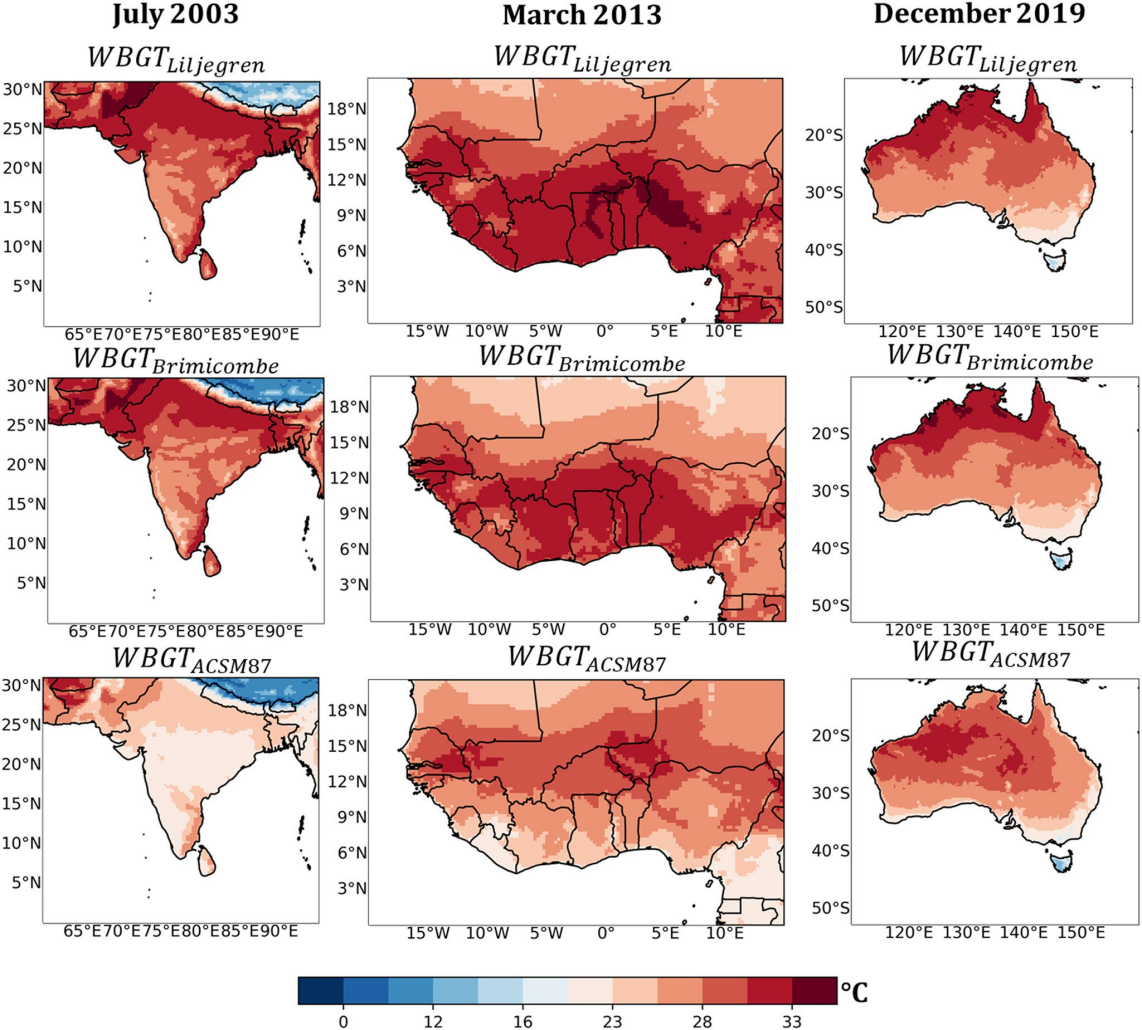
Heat stress calculated via WBGT_Liljegren_, WBGT_Brimicombe_, and WBGT_ACSM87._ Monthly mean of daily maximum Wet Bulb Globe Temperature heat stress (left to right) for the heatwaves that affected India and Pakistan in July 2003, the Western Sahel in March 2013, and Australia in December 2019. Sea area has been masked.

In July 2003, the highest values of ${WBGT}_{Liljegren}$ are over 33°C for the border of northern India and Pakistan, which is indicative of extreme heat stress in category 5 (Figure 1, left column). This pattern is well matched by ${WBGT}_{Brimicombe}$. The lowest values for both of these WBGT approximations are over the Himalayas bordering the north‐east of India, indicating no heat stress. ${WBGT}_{ACSM87}$ has lower heat stress values and only reaches 33°C, category 4.

In March 2013, the highest values of ${WBGT}_{Liljegren}$ are over 33°C and are indicative of extreme heat stress (category 5) for the north of Ghana and Nigeria (Figure 1, middle column). This pattern is broadly matched by ${WBGT}_{Brimicombe}$ although the extreme region of heat stress has slightly lower values. Similarly to the July 2003 heatwave, ${WBGT}_{ACSM87}$ has much lower values than the other approximations and only reaches at the maximum up to 33°C (category 4) in one small area. The pattern of heat stress is not well captured and ${WBGT}_{ACSM87}$ is consistently at least two heat stress categories lower than ${WBGT}_{Liljegren}$ and ${WBGT}_{Brimicombe}$ over the whole region.

For the heatwave of December 2019 in Australia, there are strikingly similar heat stress patterns for ${WBGT}_{Liljegren}$ and ${WBGT}_{Brimicombe}$ (Figure 1, right column). ${WBGT}_{ACSM87}$ also performs better for this heatwave than for the July 2003 and March 2013 heatwaves and heat stress values are close to those of ${WBGT}_{Liljegren}$. However, ${WBGT}_{ACSM87}$ again does not capture the same shape of the areas under heat stress.

**Figure 2:**
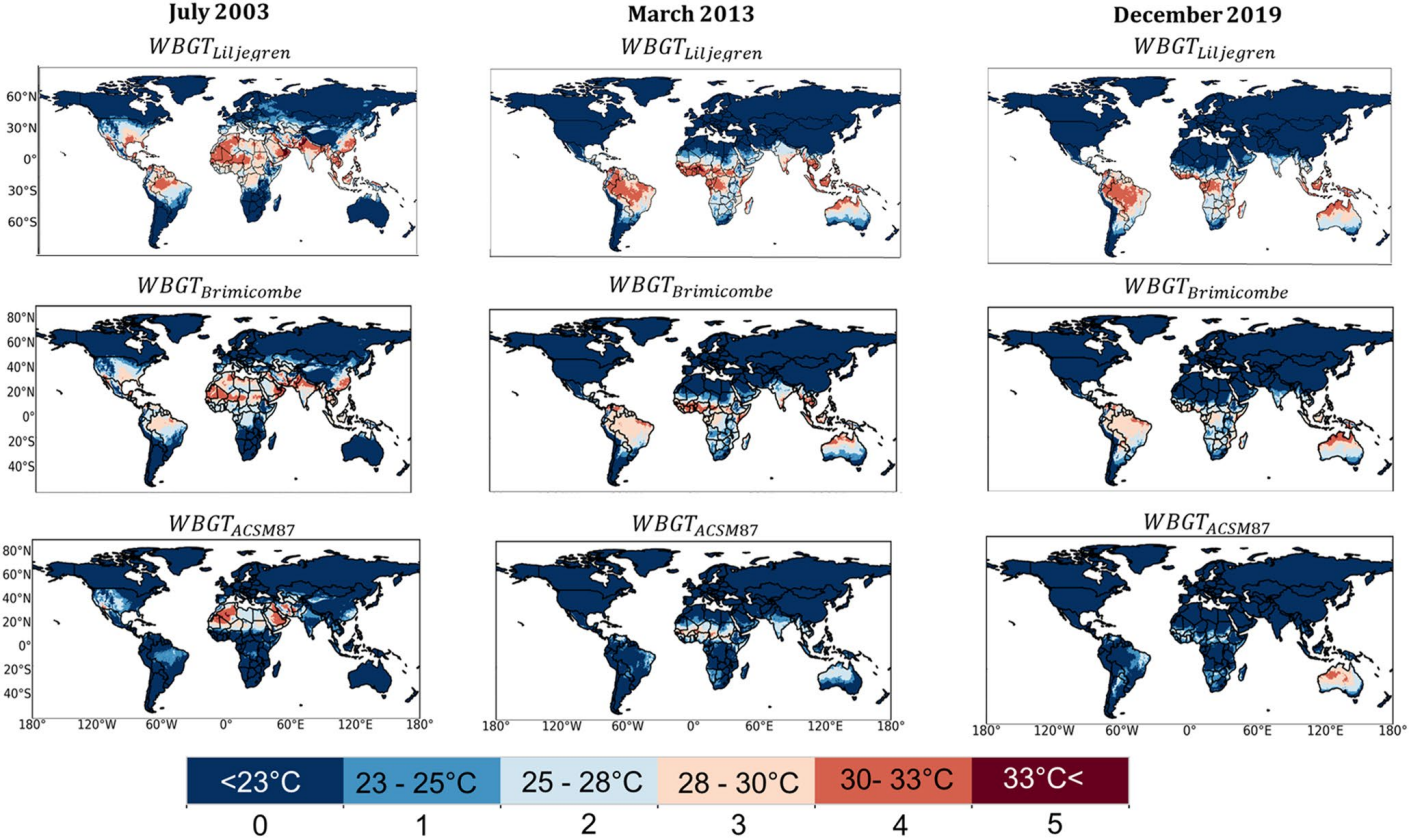
Categorical heat stress calculated via WBGT_Brimicombe_, WBGT_ACSM87_, and WBGT_Liljegren_. Monthly mean of daily maximum Wet Bulb Globe Temperature (WBGT) heat stress (left to right) for the heatwaves of July 2003, March 2013, and December 2019. Categories refer to the different levels of WBGT as indicated in Table 1. Sea area has been masked.

These similarities and differences can also be seen clearly at the global scale for each heatwave (Figure 2). ${WBGT}_{Liljegren}$ and ${WBGT}_{Brimicombe}$ values are similar worldwide in each of the 3 months considered (Figure 2), particularly focusing on parts of North Africa, southern Asia and Australia. It is however noteworthy that ${WBGT}_{Brimicombe}$ does not always capture the highest heat stress category indicated by ${WBGT}_{Liljegren}$ in South America (Figure 2). ${WBGT}_{ACSM87}$ is very different from the other approximations on a global scale. It consistently has heat stress values that are too low, sometimes three heat stress categories lower, and only captures the heat stress in some parts of Australia.

### 3.2 WBGT Approximations Anomalies

**Figure 3:**
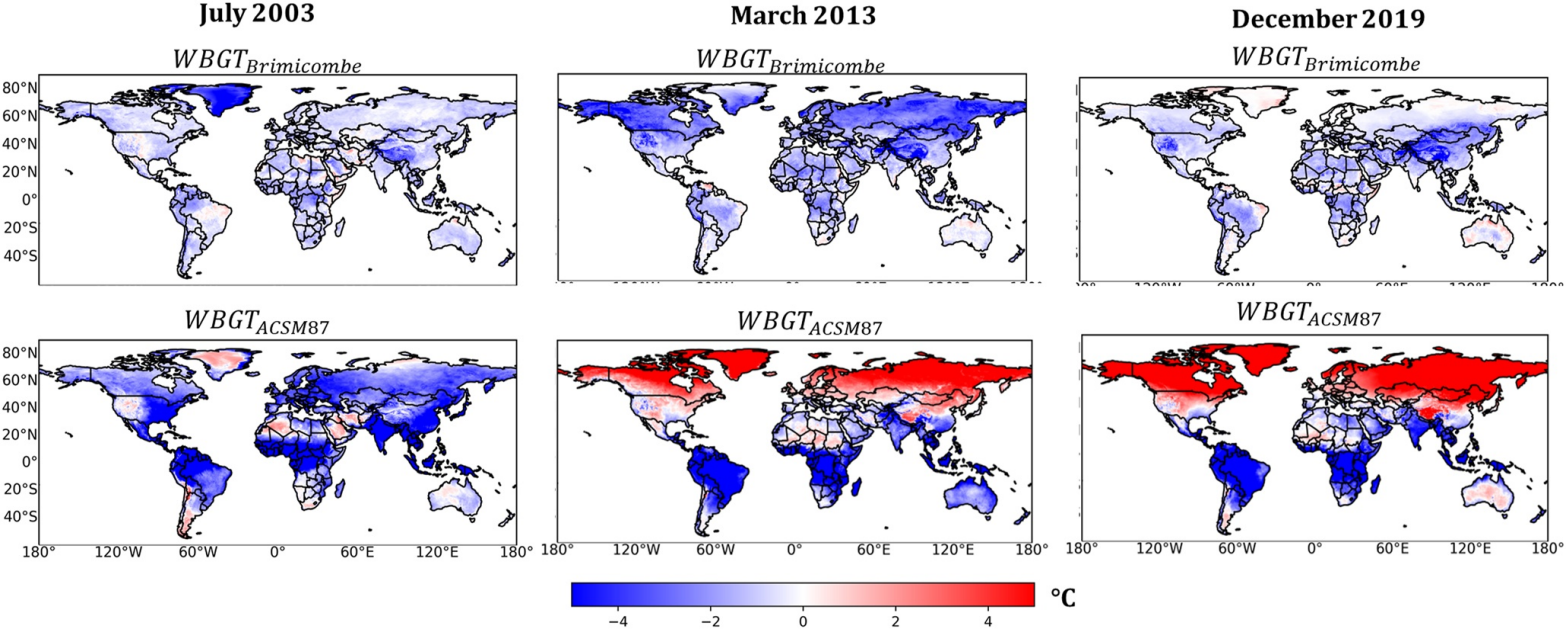
The monthly mean of the daily maxima anomalies of ${WBGT}_{Brimicombe}$ and ${WBGT}_{ACSM87}$ in comparison to WBGT_Liljegren_ for the three heatwaves considered by this study for July 2003, March 2013, and December 2019. Negative values are where WBGT_Liljegren_ has higher values than the other approximations.

Overall, the anomalies between ${WBGT}_{Liljegren}$ and ${WBGT}_{Brimicombe}$ are small, with negative anomalies indicating where ${WBGT}_{Liljegren}$ has higher values than ${WBGT}_{Brimicombe}$, the term anomaly is used to denote deviations of WBGT approximations in comparison to the current gold standard ${WBGT}_{Liljegren}$. In July 2003, WBGT_Liljegren_ has higher values than WBGT_Brimicombe_ across most of the land surface (Figure 3, left column). In addition, anomalies can be seen to be no more or less than ±2°C, except in Greenland which has anomalies of up to −4°C. In comparison, March 2013 has a similar pattern where anomalies can be seen to not be more or less than ±2°C between WBGT_Liljegren_ and WBGT_Brimicombe_ (Figure 3, middle column). However, for March 2013, more of the northern hemisphere has anomalies of −4°C, for example, in Canada and Siberia colder regions. Fewer regions experience anomalies of −4°C for December 2019, with this only present in the Himalaya into Tibet and the Canadian Rockies regions of higher elevation (Figure 3, right column). Overall across each of the three case studies, most of the land surface has anomalies of only ±2°C between WBGT_Liljegren_ and WBGT_Brimicombe_.

For WBGT_Liljegren_ in comparison to WBGT_ACSM87_ averaging across all years for the southern hemisphere, WBGT_Liljegren_ have values higher than WBGT_ACSM87_ by 4°C. In contrast, for the March 2013 and December 2019 heatwaves, the northern hemisphere has anomalies of +4°C. Anomalies are less in the Sahara desert and Australia. For the July 2003 heatwave, anomalies match with those seen in the Southern hemisphere of −4°C.

### 3.3 WBGT Approximations Correlations

**Figure 4:**
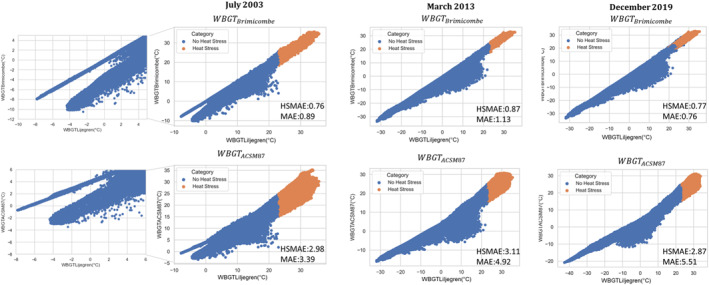
Global Grid Spatial Domain Correlation plots of ${WBGT}_{Brimicombe}$ and ${WBGT}_{ACSM87}$ in comparison to WBGT_Liljegren_ (orange indicated heat stress, i.e., Wet Bulb Globe Temperature values above 23°C). The mean absolute error (°C) for all points and just the heat stress points is indicated in each plot for the three heatwaves considered by this study (July 2003, March 2013, and December 2019).

Across both WBGT_ACSM87_ and WBGT_Brimicombe_ there is a strong linear correlation to WBGT_Liljegren_ (Figure 4). WBGT_Brimicombe_ has smaller MAE values across all three case studies than WBGT_ACSM87_, with the smallest value being 0.76°C, being on average smaller for values about the heat stress threshold. WBGT_ACSM87_ MAE values are large and range between 3.39°C and 5.51°C across the three case studies but are significantly smaller above the heat stress threshold ranging from 2.87°C to 3.11°C. WBGT_Brimicombe_ heat stress values (above 23°C, category 1 onwards) have a stronger linear relationship than across the whole distribution. In comparison, WBGT_ACSM87_ has a bigger spread in the cluster of points than WBGT_Brimicombe_ over the whole distribution.

### 3.4 WBGT Approximation Differences

**Figure 5:**
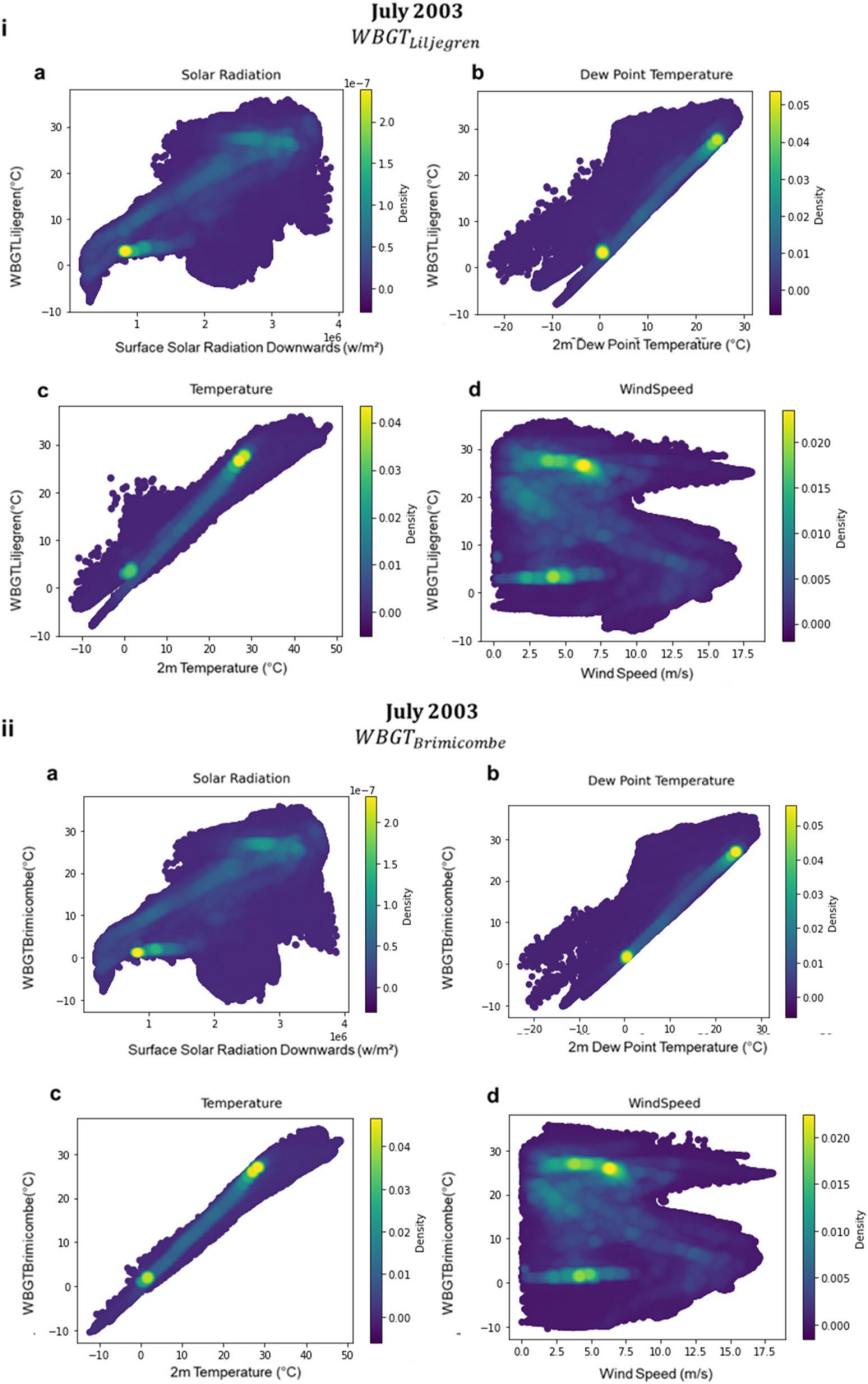
Global Grid Spatial Domain of the sensitivity of the output Wet Bulb Globe Temperature (WBGT) approximations with input variables for the July 2003 heatwave where (i) is WBGT_Liljegren_ and (ii) is WBGT_Brimicombe_ and where (a) is surface solar radiation downwards, (b) is 2 m dew point temperature, (c) is 2 m temperature, and (d) is 10 m wind speed. Input variables shown are those that are input to both WBGT approximations. Color shading denotes density of points. A similar relationship is observed for the other two heatwaves (not shown).

It has already been demonstrated that WBGT_ACSM87_ differs significantly from the other WBGT approximations presented. Therefore, here the sensitivity of only WBGT_Liljegren_ and WBGT_Brimicombe_ to key input variables is shown in more depth. Figure 5 demonstrates that broadly for both WBGT_Liljegren_ and WBGT_Brimicombe_ high solar radiation, temperature, humidity with low wind speeds lead to the highest WBGT values. In Figure 5 similarly to Figure 4 a bifurcation is seen for 2 m temperature (Figure 4) and somewhat for dew point temperature (Figure 4) with the outputted WBGT_Liljegren_. This confirms the trend is due to the WBGT_Liljegren_ Saturation Water Vapor pressure method (Figure 4)_._ As suggested in Section 2.4 this discrepancy comes from the difference in the Tw methodology, specifically from how saturation vapor pressure is calculated.

The sensitivity of WBGT_Liljegren_ and WBGT_Brimicombe_ is highly similar for solar radiation and wind speed and can be suggested to provide further evidence that Equations 2 and 6 are comparable. This is despite the potential discrepancies that were suggested in Section 2.3. This should be explored further to inform more about the inter‐dependencies of the different types of radiation. Further we find that WBGT does not have a dynamical response to wind similar to previous findings and this can be suggested to be a limitation of the heat stress index (Foster et al., 2022).

### 3.5 WBGT_Brimicombe_ Observations Comparisons

**Figure 6:**
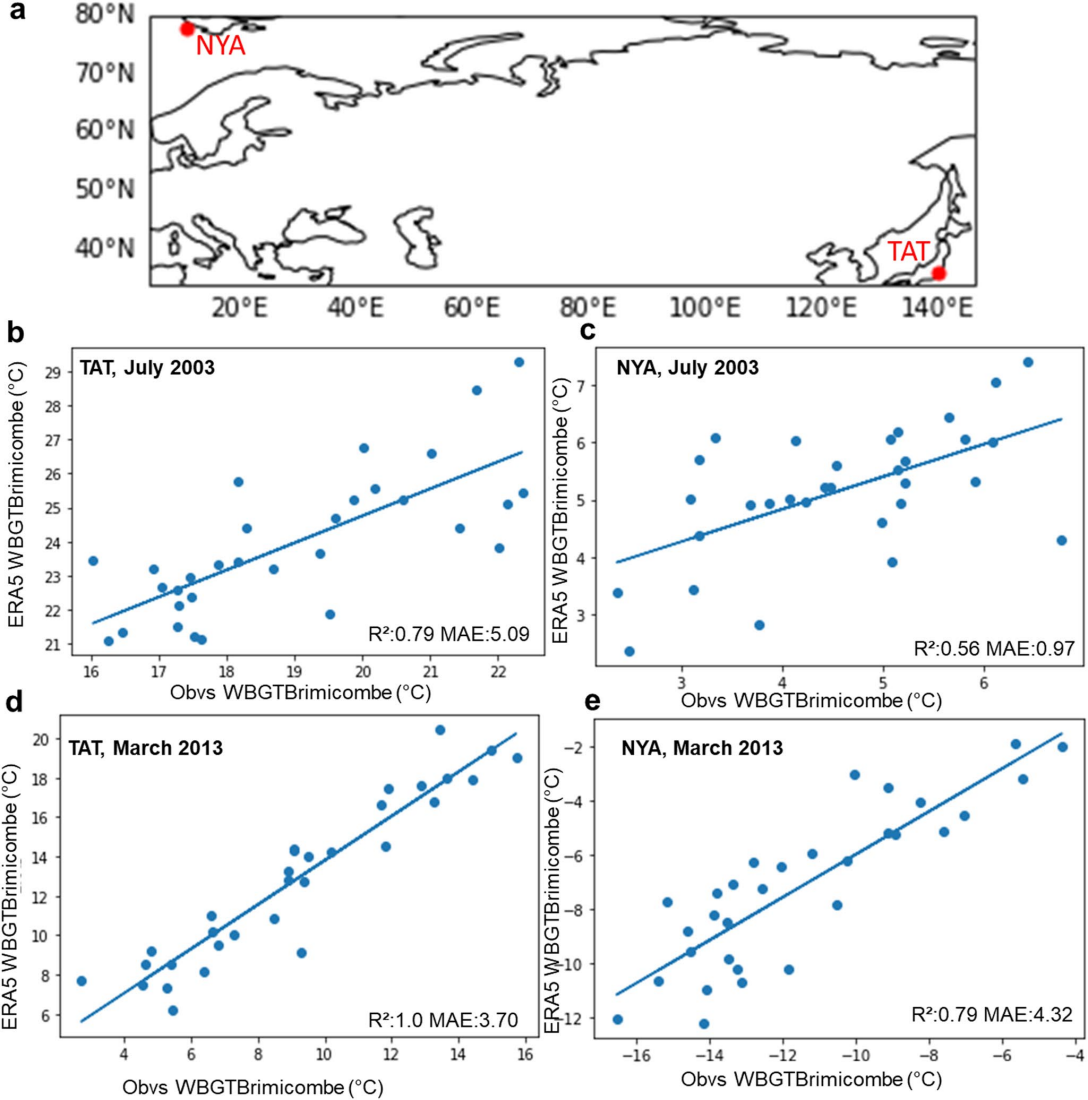
(a) The location of the observation stations NYA and TAT from the Baseline Surface Radiation Network data set to calculate observed Wet Bulb Globe Temperature using the method in thermofeel termed WBGT_Brimicombe_. (b–e) The linear relationship, *R* ^2^ and mean absolute error values for (b) TAT Daily Max July 2003, (c) NYA Daily Max July 2003, (d) TAT Daily Max March 2013, and (e) NYA Daily Max March 2013.

WBGT_Brimicombe_ for reanalysis data performs robustly in comparison to WBGT_Liljegren_ it also performs accurately compared to WBGT_Brimicombe_ observed (Figure 6). WBGT_Brimicombe_ has *R* ^2^ values between its ERA5 values and observation values of between 0.56 and 1 (Figures 6b–6e). It performs better for the TAT station (Tateno) situated in Japan, where WBGT values are higher than the NYA (Nya Långenäs) station situated in the Artic circle in Svalbard overall. MAE values range from 0.97°C to 5.06°C (Figures 6b–6e). The *R* ^2^ and error values are comparable with those seen between observed MRT and ERA5 MRT in Di Napoli et al. (2020).

In addition, when evaluating the Stull method in comparison to the Davies‐Jones method to calculate WBT for March 2013 in the observed data small differences are observed (Table 2). The biggest MAE value is 1.43°C in WBT for NYA decreasing to 1 C in WBGT. The least significant *R* ^2^ value is 0.61 for WBT for TAT. It therefore can be suggested that using Stull in comparison to Davies‐Jones makes no substantial difference in the resulting WBGT.

**Table 2:** The Mean Absolute Error and *R* ^2^ Values Between Stull Wet Bulb Temperature (WBT) and Davies‐Jones WBT and When They Are Subsequently Used to Calculate Wet Bulb Globe Temperature for Two Sets of Observations Taken During March 2013

*Station*	*Wet bulb temperature*		*Wet bulb globe temperature*	
	Mean absolute error	*R* ^2^	Mean absolute error	*R* ^2^
TAT 2013	0.808	0.611	0.56	0.81
NYA 2013	1.431	0.898	1	0.94

## 4 Discussion

### 4.1 Why Another WBGT Approximation?

We demonstrate that ${WBGT}_{Brimicombe}$ is a useful approximation of WBGT. As discussed in Section 2.4 and supported by the results in Section 3.4, ${WBGT}_{Brimicombe}$ is a beneficial method to use in the place of ${WBGT}_{Liljegren}$ for gridded data sets and numerical weather prediction services. Comparisons between ${WBGT}_{Brimicombe}$ and ${WBGT}_{Liljegren}$ show only small differences (a difference of 1 heat stress category and a MAE of between 0.76°C and 1.13°C) across the case studies considered. ${WBGT}_{Brimicombe}$ reanalysis has at most an MAE value of 5°C in comparison to it being observed (Figure 6). ${WBGT}_{Brimicombe}$ performs with the least accuracy in cold climates such as Greenland and at higher altitudes such as the Tibetan Plateau, regions that are not highly populated and are cold which is outside the scope of a heat stress index (Figure 3). As such, it has been shown with confidence that ${WBGT}_{Brimicombe}$ can be considered an accurate approximation of WBGT (Figures 1, 2, 3, 4, 5, 6).

There are many approaches to calculating the WBGT and these derive from the fact that measurements from globe and wet bulb thermometers are not widely available (Dally et al., 2018; Lemke & Kjellstrom, 2012; Lima et al., 2021; Orlov et al., 2020; Yengoh & Ardö, 2020). Unless measurements come from these instruments and provide all the input parameters required in Equation 1, all approaches to calculate the WBGT are approximations, which are wide ranging in accuracy, a wide scale observation study, in terms of both weather and physiological observations would therefore be beneficial. This research, however, clearly demonstrates that the approximation by the American college of sports medicine, ${WBGT}_{ACSM87}$, is not an accurate indication of WBGT and recommends that it is not used for a like‐for‐like approximation. This finding is in agreement with current literature on the topic (Chen et al., 2019; Grundstein & Cooper, 2018; Kong & Huber, 2021; Lemke & Kjellstrom, 2012; Lima et al., 2021; Orlov et al., 2020; Yengoh & Ardö, 2020).

Previous research has suggested that the approximation by Davies‐Jones (2008) is a more accurate approximation of natural WBT than the approximation by Stull (Buzan et al., 2015). However, the results presented here demonstrate the accuracy of the ${WBGT}_{Brimicombe}$ results and the similar sensitivity of this approximation using Stull (2011) in comparison to ${WBGT}_{Liljegren}$ and observed calculations of ${WBGT}_{Brimicombe}$. Further, for observation data it has been shown by this study that there is no more than a 1°C MAE between a WBGT output using Davies‐Jones (2008) in comparison to Stull (2011). In addition, and of particularly practical relevance, the approximation by Stull (2011) is not iterative and therefore easier to use and more readily scalable than the Davies‐Jones approximation. ${WBGT}_{Brimicombe}$ was developed for gridded data sets from numerical weather prediction data sets and is as accurate as ${WBGT}_{Liljegren}$ whilst removing the need for complicated iterative convergence methods that can practically take a long time to run and are not readily designed for gridded data. Given all of this evidence it is unnecessary to assess Davies‐Jones further by this study.

### 4.2 How Useful Are Set Thresholds for WBGT?

WBGT_ACSM87_ was found to be significantly lower than ${WBGT}_{Liljegren}$ for heat stress categories and overall is not an accurate indication of WBGT heat stress risk (as per Kong and Huber (2021)). This could be of particular disadvantage to the health sector where thresholds are often used to identify life‐threatening conditions or to recommend heat‐suitable workloads (Budd, 2008; Chen et al., 2019; Jendritzky et al., 2012; Zare et al., 2019).

In this study, it is demonstrated that ${WBGT}_{Brimicombe}$ can use the same thresholds to indicate heat stress as ${WBGT}_{Liljegren}$ with these being meaningful values for hazard preparedness. The deliberate decision is taken to use heat stress categories for WBGT as set out by Jacklitsch et al. (2016), where the highest value of 33°C has been shown to be a critical level for heat stress illnesses and to correlate with an increase in hospital admissions and mortality (Cheng et al., 2019). Many studies assessing heat stress and extreme heat are now making use of percentiles compared to a climate (Guigma et al., 2020; Heo et al., 2019) or a standard deviation compared to average conditions (Harrington & Otto, 2020). Whilst we acknowledge that heat indexes and their studies, as the present one, often still do not take into account acclimatization and that 26°C will not be experienced the same by someone in the UK in comparison to Australia (Buzan & Huber, 2020; Nazarian & Lee, 2021), we see the categorical approach as fundamental to heat hazard preparedness. We support more research into acclimatization and how to best model this with heat stress thresholds and health outcomes in mind.

### 4.3 The Use of WBGT in Weather Forecasting

The WBGT is widely used across sectors. Our approach to the WBGT has been validated in its component parts, namely in the globe thermometer temperature and the wet bulb thermometer temperature (De Dear, 1987; Guo et al., 2018; Stull, 2011). It has been demonstrated for the first time (Section 3.4) that the *T*_g_ method of ${WBGT}_{Brimicombe}$ and ${WBGT}_{Liljegren}$ are comparable. Going forward this could be used to inform more about radiation. In addition, it is designed for easy integration into operational weather prediction outputs and for use with gridded data sets, with a view to forecast heat stress and heatwaves on a global scale (Brimicombe, Di Napoli et al., 2022).

The ISO status of the WBGT makes it stand out as a heat index that is worth forecasting across multiple sectors (Heo et al., 2019). It is important to forecast WBGT to inform decisions about heat stress warnings and adaptations. There are many benefits when forecasts are made openly accessible and many factors to consider (Budd, 2008; Buzan et al., 2015; Lemke & Kjellstrom, 2012). These include: the accuracy of a WBGT approximation in comparison to the ISO observed values used in Equation 1; the robustness of thresholds in indicating heat hazards and heat stress risk levels; the appropriateness of WBGT for different climates and acclimatization levels (Ahn et al., 2022; Budd, 2008; D’Ambrosio Alfano et al., 2014). These factors also hold true for other heat indices and should be carefully considered (Ahn et al., 2022; Zare et al., 2019).

## 5 Conclusion

WBGT_Brimicombe_ has been demonstrated to be an accurate approximation of WBGT. WBGT_Brimicombe_ is within 1 heat stress category of WBGT_Liljegren_ across the land surface and in general has anomalies of no more than ±2°C for the 3 heatwave case studies here chosen. In addition, it has a strong positive correlation with WBGT_Liljegren_ and low MAE. In addition, the *T*_g_ method for WBGT_Brimicombe_ can be suggested to be equivalent to that of WBGT_Liljegren_ enhancing understanding of the relationship of different forms of radiation. Further, WBGT_Brimicombe_ has a strong linear relationship between it's observed and reanalysis data and at most an MAE of 5°C.

WBGT_ACSM87_ is not an accurate approximation of WBGT and should not be continued to be used. WBGT_ACSM87_ often has a three heat stress category difference to WBGT_Liljegren_ and it widely has anomalies of ±4°C for the three heatwave case studies chosen. Although WBGT_ACSM87_ has a strong positive correlation with WBGT_Liljegren_, it shows high MAE values.

It is hoped that by integrating WBGT_Brimicombe_ into reanalysis, climate models and forecasts, that this information would be made openly accessibly and incorporated into sectors heat warning and adaptations, providing improvements to early warning systems and adaptation policy. Finally, WBGT_Brimicombe_ is a worthy heat stress index candidate for a global forecasting early warning system and would not only be beneficial to a range of sectors but also has the real potential to save lives.

**Nomenclature**
cosθ
: Cosine of the solar zenith angle (°)
$h_{cg}$
: mean convection coefficient (W/m^2^K)
$A_{h}$
: the convective heat transfer coefficient (W/m^2^K)
$T_{a}$
: 2 m Temperature/Dry Bulb Temperature in K or °C as described
$T_{g}$
: Globe Temperature in K or °C as described
$T_{w}$
: Wet Bulb Temperature in K or °C as described
$e_{a}$
: Saturation vapor pressure of the air (kpa)
$e_{w}$
: Saturation vapor pressure of the wick (kpa)
$\alpha_{g}$
: albedo of the globe (0.05)
$\alpha_{sfc}$
: albedo of the surface (0.45)
$\varepsilon_{a}$
: the emissivity of the air (W/m^2^)
$\varepsilon_{g}$
: the emissivity of the globe (0.95)
$∆F_{net}$
: the net radiant heat flux (W/m^2^K)
*D*
: Globe Diameter 0.15 m
dsrp: downward solar radiation proportion (W/m^2^)
h
: mean convection coefficient (W/m^2^K)
ssrd: Solar Surface Radiation downwards (W/m^2^)
*T*_MRT_
: Mean Radiant Temperature in K or °C as described
*v*_a_
: 10 m wind speed m/s
*ε*
: Emissivity 0.98 that of a clothed body (Bedford & Warner, 1934; De Dear, 1987)
Nu
: Nusselt Number is the ratio of convective to conductive heat transfer (dimensionless)
P
: Surface Pressure (kpa)
Pr
: Prandtl Number is the ratio of momentum diffusivity to thermal diffusivity (dimensionless)
R
: Relative Humidity (%)
Sh
: Sherwood Number a mass transfer operation (dimensionless)
Sc
: Schmidt Number is the ratio of the kinematic viscosity to the molecular diffusion coefficient (dimensionless)
a
: is a constant of the value 0.56
e
: Saturation Water Vapor Pressure Hpa (hPa)
σ
: the Stefan‐Boltzmann constant (dimensionless)

## Conflict of Interest

The authors declare no conflicts of interest relevant to this study.

## Data Availability

ERA5 is freely accessible from Hersbach et al. (2020). WBGT_Brimicombe_ and WBGT_ACSM87_ can be calculated using *thermofeel* (Brimicombe et al., 2021, 2022) and WBGT_Liljegren_ is available on request from Kong and Huber (2021).
